# The effect of spanning external fixation on entrapped structures in tibial pilon fractures

**DOI:** 10.1007/s00590-023-03641-8

**Published:** 2023-07-11

**Authors:** Sean Thomas, Brady K. Huang, Avinaash Korrapati, Brendan O’Leary, Pradyumna Gurusamy, Ryan O’Leary, William T. Kent

**Affiliations:** 1 University of California San Diego School of Medicine, 200 West Arbor Drive MC 8894, San Diego, CA 92103 USA; 2 Division of Musculoskeletal Imaging, Department of Radiology, University of California San Diego, San Diego, CA USA; 3 Department of Orthopaedic Surgery, University of California San Diego, 200 West Arbor Drive MC 8894, San Diego, CA 92103 USA

**Keywords:** Pilon fracture, Entrapped structure, Spanning external fixation, Computerized tomography

## Abstract

**Purpose:**

Pilon fractures are often complex injuries involving severe soft tissue injury. Studies have shown pilon fractures may entrap soft tissue structures between fracture fragments. Staged fixation of pilon fractures with spanning external fixation (SEF) is important for soft tissue rest and plays an important role in the management of these injuries. While SEF has been shown to promote soft tissue rest prior to definitive fixation, no studies have shown the effect SEF has on entrapped structures (ES). The purpose of this study was to evaluate how SEF effects ES in pilon fractures.

**Methods:**

A retrospective review of 212 pilon fractures treated at our institution between 2010 and 2022 was performed. Patients with a CT scan pre-SEF and post-SEF met inclusion criteria. CTs were reviewed to characterize ES in pre- and post-SEF imaging.

**Results:**

Of the 19 patients with ES identified on CT pre-SEF, seven (36.8%) had full release of ES post-SEF and 12 (63.2%) had no release of ES. The posterior tibial tendon was the most commonly ES and remained entrapped in 62.5% of cases. Only 25% of 43-C3 fractures had release of ES post-SEF, while 100% of 43-C1 and 43-C2 fractures demonstrated complete release of ES post-SEF.

**Conclusion:**

Entrapped structures in pilon fractures are likely to remain entrapped post-SEF, with only one-third of our cohort demonstrating release. In 43-C3 patterns, if ES are identified on CT pre-SEF, surgeons should consider addressing these either through mini open versus open approaches at the time of SEF as they are likely to remain entrapped post-SEF.

## Introduction

Pilon fractures are often the result of high-energy axial load of the talus into the tibial plafond. These fractures tend to have anatomically reproducible fracture patterns, including the anterolateral (Chaput), posterolateral (Volkmann), and medial malleolar fragments [1]. Previous studies have shown that in patients with severe tibial pilon fractures, soft tissue structures, most commonly the posterior tibial tendon (PTT) and posterior tibial neurovascular bundle, may become incarcerated between these fragments [2, 3]. Recognizing the entrapment of these structures is important as it aids the surgeon in choice of surgical approach and plan for liberating these structures during fixation.

Due to a tenuous soft tissue envelope and subsequent risk of wound complications in severe tibial pilon fractures, a staged approach involving spanning external fixation (SEF) before definitive open reduction internal fixation has become the standard of care in high energy pilon fractures [4, 5]. Through ligamentotaxis, ankle SEF allows the surgeon to obtain improved length and alignment of the tibia, talus, and hindfoot, and create an improved environment for soft tissue rest. These staged protocols have demonstrated reduced rates of infection and improved outcomes when compared to immediate open reduction internal fixation [1, 4].

Although it has been established that tibial pilon fractures may entrap soft tissue structures, and that SEF allows for optimal soft tissue rest and recovery from high-energy trauma, no studies have shown how SEF effects the fate of these entrapped structures. We have encountered several patients with entrapped structures in the setting of severe tibial pilon fractures with CT scans acquired both at the time of injury and after SEF. The purpose of this study was to define how SEF effects entrapped structures in tibial pilon fractures.

## Materials and methods

After obtaining institutional review board (IRB) approval, we conducted a 12-year retrospective review from January 2010 to September 2022 of our database at a regional Level 1 Trauma Center. A waiver of informed consent was granted by our institutional review board for the conduction of this retrospective study. We performed the search on our radiology information system (RIS) using M*Modal Catalyst version 3.17.01. (M*Modal, Franklin, TX). This search included all CT examinations of lower extremities. Search terms included were “pilon” and “plafond.” Inclusion criteria were skeletally mature patients, presence of an intraarticular fracture of the distal tibia (classified as either OTA/AO 43-B or C type injuries), presence of CT scans before and after SEF, and a complete medical chart documenting definitive treatment.

**Fig. 1:**
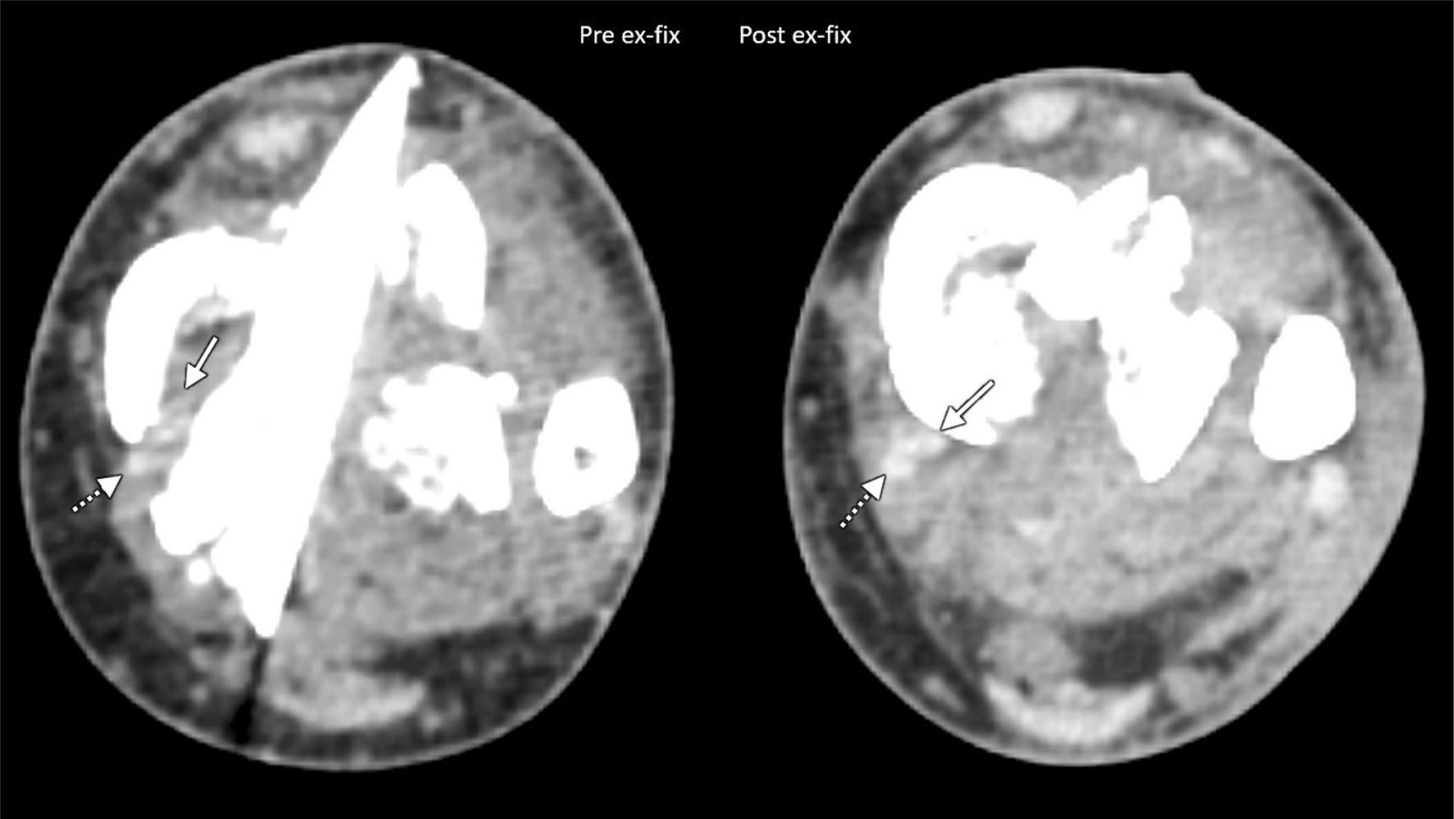
Thirty-one-year-old man who fell from the US border fence, with AO/OTA 43C3.3 pilon fracture (Ruedi/Allgower type 3). Initial axial trauma CT angiogram (left) shows entrapment of the posterior tibial tendon (solid arrow) and flexor digitorum longus tendon (dotted arrow) within the fracture. Post-SEF CT (right) shows release of both tendons

**Fig. 2:**
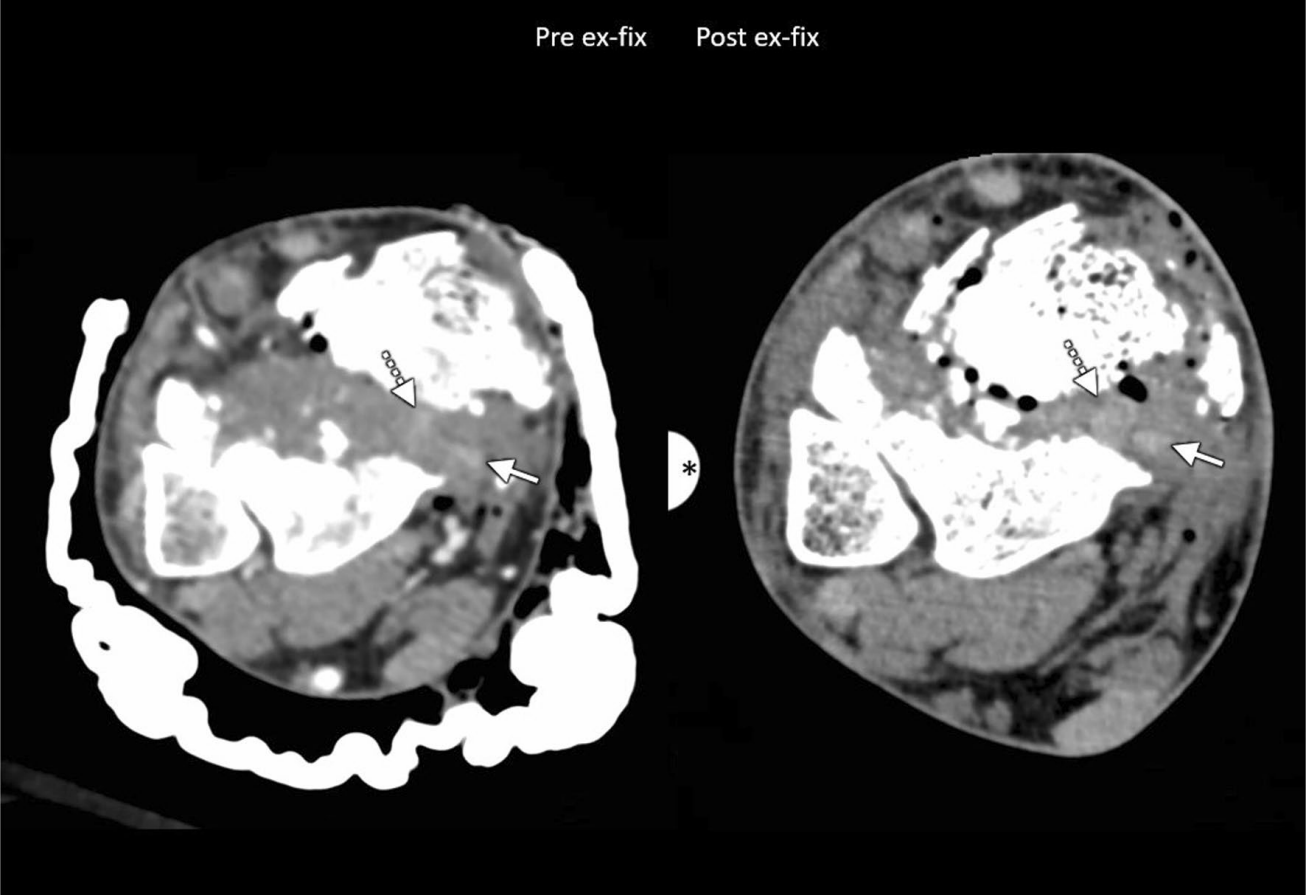
Forty-four-year-old man who fell from the US border fence, with AO/OTA 43C3.3 pilon fracture (Ruedi/Allgower type 3). Initial trauma CT angiogram (left) shows entrapment of the posterior tibial tendon (solid arrows) and flexor digitorum longus tendon (dotted arrows) within the fracture. Post-SEF CT (right) shows persistent entrapment of both tendons. External fixation device is present (asterisk)

For those patients in whom inclusion criteria was met, the pre- and post-SEF CT scans were read by a fellowship-trained musculoskeletal radiologist for presence of partial or fully entrapped structures. Identifiable structures include the PTT, flexor hallucis longus (FHL), flexor digitorum longus (FDL), anterior tibial tendon (ATT), extensor hallucis longus (EHL) and the anterior tibial artery (ATA). For this study, we defined the complete entrapment of a tendon or neurovascular structure as having the entirety of its axial width interposed between fracture fragments, and partial entrapment as having some amount of axial width outside fracture lines. A structure was noted to be completely released post-SEF if its entire axial width had been liberated from between fracture fragments (Figs. 1 and 2). If a structure was completely entrapped pre-SEF, and had a portion of its axial width equal to at least 50% released from between fragments, it was noted to be partially released.

## Results

From January 11, 2010 to September 6, 2022, we identified 657 CT exams matching the term “pilon” or “plafond”. Of those, we identified 490 scans that were true intraarticular distal tibia fractures. Within this sample, 36 (7.3%) had a CT scan performed both before and after SEF. Of these 36 cases, 19 (52.8%) had entrapped structures pre-SEF. Using AO/OTA classification, 16 (84.2%) of these 19 fractures were identified as 43-C3, 2 (10.5%) as 43-C2, and 1 (5.3%) as 43-C1. Fifteen (78.9%) fractures were the result of falls from height, 2 (10.5%) were from motorcycle collisions, 1 (5.3%) was a pedestrian versus auto injury, and 1 (5.3%) was the result of a motor vehicle collision (Table 1).

**Table 1:** Fracture mechanism, classification, and entrapped structures

Case	Mechanism of injury	AO/OTA	Entrapped structures pre-SEF (with % of axial width entrapped)	Entrapped structures post-SEF (with % of axial width entrapped)
1	Fall from height	43-C3.2	PTT (50%)	PTT (50%)
2	MCC	43-C2.3	PTT (100%), FDL (100%)	None
3	Fall from height	43-C3.2	PTT (100%), FDL (100%), FHL (25%)	None
4	Fall from height	43-C3.2	PTT (100%)	None
5	Fall from height	43-C3.2	PTT (100%)	PTT (50%)
6	Fall from height	43-C3.3	PTT (100%), FDL (100%), ATA (100%)	PTT (100%), FDL (100%)
7	Fall from height	43-C3.3	PTT (100%), FDL (100%)	PTT (100%)
8	Fall from height	43-C3.3	PTT (50%)	PTT (50%)
9	MCC	43-C3.3	PTT (100%), FDL (50%)	PTT (100%), FDL (50%)
10	Peds versus auto	43-C3.1	PTT (100%)	PTT (100%)
11	Fall from height	43-C3.3	PTT (100%)	PTT (100%)
12	MVC	43-C1.2	PTT (intra-articular)	None
13	Fall from height	43-C2.3	ATT (100%), EHL (100%)	None
14	Fall from height	43-C3.2	ATA (100%)	ATA (100%)
15	Fall from height	43-C3.3	PTT (100%), FHL (100%)	PTT (100%), FHL (100%)
16	Fall from height	43-C3.2	PTT (50%)	None
17	Fall from height	43-C3.3	PTT (100%), FHL (50%)	None
18	Fall from height	43-C3.2	PTT (100%)	PTT (100%)
19	Fall from height	43-C3.2	ATA (50%)	ATA (50%)

*SEF* spanning external fixation, *PTT* posterior tibial tendon, *MCC* motorcycle collision, *FDL* flexor digitorum longus, *MVC* motor vehicle collision, *FHL* flexor hallucis longus, *ATT* anterior tibialis tendon, *EHL* extensor hallucis longus, *ATA* anterior tibial artery

Within this cohort of 19 patients with entrapped structures pre-SEF, seven (36.8%) demonstrated complete release of entrapped structures, two (10.5%) had partial release of entrapped structures, and ten (52.6%) had no release of entrapped structures following SEF. The PTT was interposed pre-SEF in 16 (84.2%) cases, the FDL in five (26.3%) cases, the FHL and ATA each in three (15.8%) cases, and the ATT and EHL each in one (5.3%) case (Table 2). The PTT underwent full release post-SEF in six (37.5%) cases, was partially released in two (12.5%) cases, and remained entrapped in eight (50%) cases. The FDL was fully released in three (60%) cases, the FHL in two (66.7%) cases, and the ATT and EHL each in one (100%) case. Evaluation of entrapment of the ATA required post-SEF CT angiography, which was only obtained in 2/3 fractures with ATA entrapment. The ATA remained entrapped post-SEF in both cases (2/2) (Table 2).

**Table 2:** Status of entrapped structures pre- and post-spanning external fixation

Entrapped structure	Number entrapped pre-SEF (of 19 fractures)	Full release post-SEF	Partial release post-SEF	No release post-SEF	Percent with full or partial release post-SEF (%)
PTT	16 (84.2%)	6 (37.5%)	2 (12.5%)	8 (50%)	50
FDL	5 (26.3%)	3 (60%)	0	2 (40%)	60
FHL	3 (15.8%)	2 (66.7%)	0	1 (33.3%)	66.7
ATT	1 (5.3%)	1 (100%)	0	0	100
EHL	1 (5.3%)	1 (100%)	0	0	100
ATA	3* (18.8%)	0	0	2 (100%)	0

*SEF* spanning external fixation, *PTT* posterior tibial tendon, *FDL* flexor digitorum longus, *FHL* flexor hallucis longus, *ATT* anterior tibialis tendon, *EHL* extensor hallucis longus, *ATA* anterior tibial artery

^*^One instance of ATA entrapment could not be evaluated post-SEF due to lack of CT angiogram

Nine patients had isolated PTT entrapment pre-SEF. Following SEF, three (33.3%) underwent complete release, one (11.1%) had partial release, and five (55.6%) remained fully entrapped. In the three fractures with PTT and FDL entrapment, one (33.3%) demonstrated complete release of all structures (Fig. 1), one (33.3%) had full release of the FDL with the PTT remaining entrapped, and one (33.3%) had no release of either the FDL or PTT (Fig. 2). In the two fractures with both PTT and FHL entrapment pre-SEF, one (50%) demonstrated complete release of entrapped structures post-SEF, while the other had all structures remained entrapped post-SEF. In the one patient with PTT, FDL, and FHL entrapment, all three tendons were completely released post-SEF. One patient had entrapment of the ATT and EHL pre-SEF, with both structures released post-SEF. Two fractures displayed isolated ATA entrapment, and neither had observed release post-SEF. One case demonstrated entrapment of the ATA along with the PTT and FDL. In this case, ATA position could not be evaluated post-SEF due to lack of appropriate post-contrast imaging. The PTT and FDL remained fully entrapped post-SEF (Table 1).

Full release of entrapped structures post-SEF was observed in 25% of 43-C3 fractures and in 100% of 43-C1 and 43-C2 fractures, a statistically significant difference (*p* = 0.01) (Table 3). The rate of PTT release post-SEF was similar between fractures with isolated PTT entrapment (33.3%) and non-isolated PTT entrapment (43.9%) (*p* = 0.70).

**Table 3:** Status of entrapment of pre- and post-spanning external fixation by fracture classification

Fracture classification	*n*	Full release post-SEF	Partial release post-SEF	No release post-SEF
43-C1	1	1 (100%)	0	0
43-C2	2	2 (100%)	0	0
43-C3	16	4 (25%)	2 (12.5%)	10 (62.5%)

*SEF* spanning external fixation

## Discussion

Tibial pilon fractures may involve a broad spectrum of injury and soft tissue compromise [1, 2]. In severe cases, significant displacement between fracture fragments at the level of the tibial plafond may allow for entrapment of the adjacent posteromedial soft tissue bundle [3]. Characterization of pilon fracture morphology, associated injury to surrounding structures, and planned surgical approach rests on the use of CT [2, 3, 6].

The utility of CT in the identification of entrapped structures in pilon fractures has been well-documented [2, 6, 7]. Studies by Tresley et al. and Crim et al. found the rate of entrapped structures in pilon fractures to be 24% and 21.5%, respectively [6, 8]. Among fractures with entrapped structures, Tresley et al. found the PTT to be entrapped most frequently in 86.4% of cases, the FDL in 36.6% of cases, and the FHL in 9.1% of cases [8]. In line with these findings, we report PTT entrapment in 84.2% of cases, FDL entrapment in 26.3% of cases, and FHL entrapment in 15.8% of cases. In addition to these posteromedial structures, we identified entrapment of the ATA in 15.8% of cases, and entrapment of the ATT and EHL each in 5.3% of cases (Table 2).

While previous reports detail the incidence of entrapped structures and verify the use of CT in their identification, they do not describe how SEF effects entrapped structures. In a review of the rate of posteromedial structure entrapment in pilon fractures, Fokin et al. identified eight patients with pre- and post-SEF CT scans, one (12.5%) of which showed evidence of release post-SEF [3]. In a smaller cohort, Eastman et al. identified three patients with pre- and post-SEF CT exams, one (33.3%) of which underwent release post-SEF [2]. Understanding how SEF impacts the fate of entrapped structures is useful in surgical planning and provides insight into how this intervention may affect soft tissue involvement in pilon fractures. We report the largest sample size to date evaluating the impact of SEF on entrapped structures in pilon fractures.

In our sample of 19 patients with both pre- and post-SEF CT scans, we observed full release of entrapped structures in only 36.8% of cases. The PTT was the most commonly entrapped structure, and was fully released from between fracture fragments in 37.5% (6/16) of cases. There were nine cases of isolated PTT entrapment and seven cases of PTT entrapment alongside other structures (Table 1). Of note, the rate of full release of an isolated entrapped PTT (33.3%) did not vary significantly from cases of non-isolated PTT entrapment (42.9%) (*p* = 0.70). This suggests that release of the PTT post-SEF is not affected by the presence of other entrapped structures. In regard to other posteromedial structures, the FDL and FHL were fully released post-SEF in 60% and 66.7% of cases, respectively (Table 2). Though the sample size for these structures is limited, we also report complete release of the ATT and EHL in every instance, while the ATA did not undergo release in both cases where CT angiography was obtained (Table 2).

Previous studies have identified that entrapment of posteromedial structures is more likely to occur in 43-C3 fractures when compared to 43-C1 and 43-C2 fractures [2, 3]. In addition to reporting a higher percentage of 43-C3 fractures in our cohort, we also show that 43-C3 fractures are less likely to undergo full release of entrapped structures post-SEF (25%) when compared to 43-C1 and 43-C2 fractures (100%) (*p* = 0.01). As noted by Eastman et al. higher degrees of displacement in 43-C3 fractures create more room for the entrapment of posteromedial structures [2]. This increased displacement between fracture fragments in 43-C3 fractures appears to preclude release of structures, leading to higher rates of entrapment post-SEF when compared to 43-C1 and 43-C2 fractures.

Entrapment of posteromedial structures can result in worse clinical outcomes for patients, and early identification is important. Failure to release entrapped tendons at the time of definitive fixation results in prolonged entrapment of structures and is associated with long-term stiffness, limited range of ankle dorsiflexion, extensive soft tissue compromise requiring coverage, and equinovarus and claw toe deformity [9–13]. While the implications of prolonged tendon entrapment have been well-documented, the clinical benefit of releasing entrapped structures at the time of SEF has not been described. It can be reasonably assumed that early release of entrapped structures would have a positive effect on patient outcomes, but long-term outcome studies are needed.

Though the clinical benefit of addressing entrapped structures at time of SEF has not been determined, understanding the location of entrapped structures post-SEF is still important for surgical planning, as the presence of an entrapped structure may influence choice of surgical approach for definitive fixation. If an entrapped structure cannot be released through an anteromedial or anterolateral approach, an additional posteromedial approach may be necessary [2].

The limitations of this study include it being retrospective in nature, and it is therefore subject to some limitations in data collection. Many of our patients were referred from outside institutions and received imaging and treatment prior to transfer to our facility. All available medical records were reviewed to ensure accurate collection of data. In addition, entrapped structures in pilon fractures are rare, and it is not our standard practice to order imaging both before and after SEF. Our sample size was therefore limited, and future studies including more patients would improve our understanding of the impact of SEF on entrapped structures. Despite these limitations, this study presents the largest sample size to date analyzing the impact of SEF on entrapped structures in pilon fractures.

In conclusion, we report that entrapped structures in tibial pilon fractures are likely to remain entrapped post-SEF. In patients with both pre- and post-SEF imaging, full release of entrapped structures was documented in only one-third of cases. Structures are more likely to be released post-SEF in simple complete articular fracture patterns (43-C1 and 43C-2) when compared to 43-C3 patterns. If entrapped structures are identified on CT pre-SEF in 43-C3 injuries, surgeons should consider addressing these either through mini open versus open approaches at the time of SEF, as these structures are likely to remain entrapped post-SEF. The clinical implications of tendon release at the time of SEF have not been elicited, though a case can be made for addressing entrapped structures acutely. While it is not the standard to obtain both pre- and post-SEF CT scans, there are often instances when pre-SEF is available for other reasons. If available, it is important to note the presence of entrapped structures, and if a C3 pattern, the entrapped structure is likely to remain entrapped after SEF. In addition, if the ATA is identified as entrapped in the fracture, it will likely remain entrapped, and one should consider an open approach to address this in a more timely fashion.

## References

[1] Topliss CJ, Jackson M, Atkins RM (2005). Anatomy of pilon fractures of the distal tibia. J Bone Jt Surg Br.

[2] Eastman JG, Firoozabadi R, Benirschke SK (2014). Entrapped posteromedial structures in pilon fractures. J Orthop Trauma.

[3] Fokin A, Huntley S, Summers SH (2016). Computed tomography assessment of peroneal tendon displacement and posteromedial structure entrapment in pilon fractures. J Orthop Trauma.

[4] Blauth M, Bastian L, Krettek C (2001). Surgical options for the treatment of severe tibial pilon fractures: a study of three techniques. J Orthop Trauma.

[5] Patterson MJ, Cole JD (1999). Two-staged delayed open reduction and internal fixation of severe pilon fractures. J Orthop Trauma.

[6] Sirkin M, Sanders R, DiPasquale T (1999). A staged protocol for soft tissue management in the treatment of complex pilon fractures. J Orthop Trauma.

[7] Assal M, Adrien R, Richard S (2015). Strategies for surgical approaches in open reduction internal fixation of pilon fractures. J Orthop Trauma.

[8] Crist BD, Khazzam M, Murtha YM (2011). Pilon fractures: advances in surgical management. J Am Acad Orthop Surg.

[9] Crim J, Enslow M, Smith J (2013). CT assessment of the prevalence of retinacular injuries associated with hindfoot fractures. Skelet Radiol.

[10] Ho RT, Smith D, Escobedo E (2001). Peroneal tendon dislocation: CT diagnosis and clinical importance. Am J Roentgenol.

[11] Tresley J, Subhawong TK, Singer AD (2016). Incidence of tendon entrapment and dislocation with calcaneus and pilon fractures on CT examination. Skelet Radiol.

[12] Colomb E, Muscatelli S, Morash JG (2021). Irreducible fractures and dislocations of the ankle associated with entrapment of the posterior tibial tendon within the tibiofibular interosseous space: a case series and literature review. Foot Ankle Orthop.

[13] Thoreau L, Kaminski L, Putineanu DC (2019). Irreducible ankle fracture dislocation due to posterior tibialis tendon interposition: Diagnostic and clues for early management—a case report. Trauma Case Rep.

